# A randomized, 12-month, parallel-group trial testing the effects of a cognitive behavioral healthy lifestyle intervention as an adjunct to standard trauma therapy among adults with PTSD: the I-CHAT study protocol

**DOI:** 10.3389/fpsyg.2025.1685371

**Published:** 2025-11-27

**Authors:** Jeffrey L. Kibler, Abigail Owney, Carolina Rosado, Shalynn Murphy, Maria Magdalena Llabre, Karla Patricia Molina Valenzuela, Claudia Ocholski, Mindy Ma

**Affiliations:** 1 College of Psychology, Nova Southeastern University, Fort Lauderdale, FL, United States; 2 Department of Psychology, University of Miami, Coral Gables, FL, United States

**Keywords:** PTSD, health behaviors, intervention, healthy lifestyle, physical activity, sleep

## Abstract

Research has indicated strong associations between posttraumatic stress and cardiovascular disease (CVD) risk. Individuals with posttraumatic stress disorder (PTSD) tend to show patterns of elevated CVD risk earlier in life than the general population. The need for developing effective interventions for CVD risk-reduction in PTSD is increasingly evident. The present paper outlines the theoretical background and methodological details for the protocol of an ongoing NHLBI-funded longitudinal study (entitled *Investigating Cardiac Health of Adults with Trauma*) to test the effects of a health behavior intervention as an adjunct to standard trauma therapy in PTSD. The health behavior intervention addresses CVD-related health behaviors (physical activity, nutrition, sleep, and stress). Participants are randomized to the health behavior intervention plus standard trauma therapy condition or a standard trauma therapy control group. Outcome assessments are conducted before and after the 12-week intervention program in the experimental group, and 12 weeks apart for the control group, as well as at 6-month and 12-month follow-up time points. The outcomes include laboratory measures of CVD risks/markers (e.g., endothelial function, arterial stiffness, lipids, blood pressure), actigraphy-based measures of physical activity and sleep, and standardized self-report measures of sleep, physical activity, nutrition, stress, and psychological functioning (e.g., PTSD symptoms, depressive symptoms). The study findings will provide valuable data on the effectiveness of the health behavior intervention in producing predicted changes in the target CVD-related behaviors/markers.

## Introduction

Strong relationships between PTSD and cardiovascular disease (CVD) risk have been identified, with numerous studies indicating those with posttraumatic stress disorder (PTSD) are at greater CVD risk, earlier in life (Browne et al., 2021; Kibler et al., 2018; Reis et al., 2023; Seligowski et al., 2024; Smith et al., 2020a; Sumner et al., 2020). The need for developing effective interventions for CVD risk-reduction in PTSD is increasingly evident. Compared to the cumulative evidence concerning elevated CVD risk in PTSD, relatively little research has addressed CVD risk-reduction in this population (Bourassa et al., 2021; Farr et al., 2014; Krantz et al., 2022; Li et al., 2024; National Heart Lung and Blood Institute, 2018; Scherrer et al., 2020). One study revealed that Veterans whose PTSD symptoms improved while treated in Veterans Affairs (VA) PTSD specialty clinics did not show significant improvement in CVD risks (Scherrer et al., 2020). Thus, adjunctive treatments, such as health behavior interventions, may be necessary as supplements to traditional psychotherapy for PTSD to reduce CVD risks. Health behavior interventions are needed that specifically address unique aspects of PTSD symptom presentation, which serve as barriers to healthy behaviors (e.g., avoidance of physiological arousal/activation, nightmares/sleep disruption, and cognitive responses to stress) and may not be adequately targeted with standard mental health treatments (Farr et al., 2014; Li et al., 2024; National Heart Lung and Blood Institute, 2018; Scherrer et al., 2020).

Heightened awareness or fear of bodily arousal symptoms, such as increased heart rate or shortness of breath, have been associated with less physical activity and lower ratings of exercise motivation in PTSD (Crombie et al., 2023; Hall et al., 2015; Harte et al., 2015). In conjunction with anxiety reactions, the fear of bodily arousal can be conceptualized as anxiety sensitivity, which is commonly reported among those with PTSD (Asmundson and Stapleton, 2008; Crombie et al., 2023; Stanley et al., 2017). Anxiety sensitivity may lead to avoidance of exercise (Olthuis et al., 2023). Conversely, there is experimental evidence that engaging in physical activity can decrease anxiety sensitivity (Broman-Fulks et al., 2004; Crombie et al., 2023; Hall et al., 2015), thereby reversing the negative cycle of avoidance at least temporarily. These effects are theorized to occur through exposure and desensitization to internal arousal cues (Broman-Fulks et al., 2004). Potential fear of arousal symptoms may underscore the need to begin slowly when implementing physical activity for individuals with PTSD who have been sedentary. Research and health behavior theories support the notion that abrupt increases in activity are not as likely to be maintained (Sabourin et al., 2011). Such abrupt changes may be more difficult when one has hypersensitivity to arousal (Sabourin et al., 2011). Therefore, our experimental intervention was designed to be sensitive to the specific needs of participants with PTSD. Participants report positive responses to being able to work at their own pace and feel supported by interventionists who “met them where they are” regarding physical activity. In addition, our strategy of having interventionists walk with participants during designated sessions allows the interventionist to address perceptions of arousal in the moment, and to encourage continued exposure and desensitization while providing support. Hall et al. (2020) indicated that increasing physical activity is feasible and can enhance aerobic performance and metabolic measures among older military Veterans with PTSD.

There is evidence that unhealthy lipid levels and higher weight are more prevalent among individuals with PTSD (Bharti et al., 2022; Kibler et al., 2018; Rossi and Isnard, 2023); 75–85% of adults with PTSD meeting criteria for overweight and 45–50% for obesity. These findings are consistent with the hypothesis that over-eating and consumption of high-fat foods may contribute to CVD in PTSD (Hirth et al., 2011). Although lipid levels are determined by additional physiological factors beyond eating, research of this issue generally indicates behavioral factors such as food choices and physical activity confer at least an interactive effect with genes and medical factors (Vinson et al., 2008). A recent study found that elevated triglycerides in PTSD were associated with greater blood pressure levels (Bhargava et al., 2024). One of the key theoretical concepts implicated in the eating behavior of those with PTSD is that eating is a mechanism for coping. Across trauma types, binge eating to cope with trauma-related stressors has been observed (Harrington et al., 2010; Tagay et al., 2014). Binging may represent a form of dissociation or a way of focusing attention away from distressing thoughts and negative emotion (Huston et al., 2019). Another phenomenon related to regulation of eating in PTSD is that abused individuals often have a disrupted sense of self-worth and attractiveness. This can, in turn, manifest in diminished self-care, such as overeating and physical inactivity (Gustafson and Sarwer, 2004; Wolf and Elklit, 2018).

Recurring trauma-related nightmares in PTSD and consequent behavioral conditioning of sleep avoidance are disruptive, prolonging sleep latency and contributing to irregular bedtimes (Lamarche and De Koninck, 2007; Schumm et al., 2023). Moreover, these types of disruptions in sleep have relevance for CVD risk/outcomes (Covassin and Singh, 2016; Huang et al., 2020; Makarem et al., 2019; Scherrer et al., 2020). The study by Scherrer et al. (2020) demonstrated that sleep disorders mediated PTSD/CVD associations. Thus, sleep interventions may enhance CVD risk-reduction efforts (Javaheri et al., 2020; Makarem et al., 2019). Psychotherapies for PTSD have not typically addressed sleep problems directly or systematically, and sleep outcomes following therapy have not generally been assessed (Galovski et al., 2009; Pruiksma et al., 2016). The limited number of CBT interventions that have focused specifically on sleep in PTSD have resulted in improved sleep (Krakow et al., 2001; Moore and Krakow, 2007). Therefore, additional research on this area of treatment may further inform the potential for health behavior intervention to impact health in PTSD. Relationships of sleep with physical activity and stress (Eid et al., 2021; Zhu et al., 2021) may help in explaining the potential role of sleep in associations between PTSD and CVD risk (Scherrer et al., 2020).

Stress-related and emotional factors have been identified as an early candidate mechanism for PTSD/CVD relationships, with various responses to acute and chronic stress being implicated in this relationship (Smith et al., 2020a,b). For example, PTSD severity was shown to be related to delayed cardiovascular recovery following a general (not trauma-related) laboratory oral speaking stressor (Kibler, 2018). Delayed cardiovascular recovery in the lab has been associated with greater carotid intima-media thickness, as well as elevated resting blood pressure measured 3 years after lab assessment of recovery (Steptoe et al., 2006; Steptoe and Marmot, 2005). Perceived threat (a pervasive maladaptive cognitive response in PTSD) mediated these physiological disruptions (Kibler, 2018). Research has identified cognitive appraisals, including perceived threat, as key factors to address in PTSD (Iverson et al., 2015). Although the implications of cognitive appraisals for health behaviors in PTSD have not been adequately researched, relationships of appraisals to stress are evident, and Lazarus and colleagues' Cognitive Appraisal Theory (Lazarus and Folkman, 1984) suggests that the types of disrupted cognitions evident in PTSD may interfere with the pursuit of healthy behaviors such as physical activity (Folkman et al., 1986). Interventions to address cognitive appraisals may reduce stress-related CVD risk in PTSD by enhancing the ability to cognitively cope with stressors and engage in healthy behaviors.

The purpose of the present paper is to detail our approach to the study protocol, focusing on the scientific rationale and methodological details. It is hypothesized that the healthy lifestyle intervention will result in greater improvement in vascular function (i.e., endothelial function, arterial stiffness), physical activity, nutrition, sleep, and stress, above and beyond the effects of standard trauma therapy.

## Methods

### Participants

We plan to randomize a total of 212 participants, ages 18 and over, to the two study conditions. Anticipated demographics of the overall sample, based on current data from proposed recruitment sites and prior studies recruiting from the PTSD population, are ages 37 ± 10 (mean ± SD), approximately 72% female, avg. of 12th grade education ± 3 years, and race/ethnicity composed of 64% Caucasian, 21% Hispanic-White, 10% African-American, 3% Caribbean Black, and 2% Hispanic-Non-White. Adults ages 18 and older are recruited from the community, local clinics, and via online advertisements. Criteria for inclusion are: PTSD or subthreshold PTSD [evidenced by ≥ 4 PTSD symptoms (including ≥ 1 re-experiencing symptom) endorsed as 2 or higher (moderately bothersome) on the Posttraumatic Stress Checklist (PCL-5)], the presence of at least one health risk (low physical activity, nutrition does not meet standard recommendations, overweight, sleep disruption), and ability to exercise at a moderate level. Concurrent trauma-focused psychotherapy is required through the 12-week intervention phase of the project and is provided by the project if the participant is not already receiving trauma-focused therapy in the community. On an ongoing basis, the project coordinator verifies the therapy and frequency. Although chronic illnesses are not exclusionary, medications for controlling lipids or blood pressure (e.g., beta blockers, diuretics, ACE inhibitors, statins, PCSK9 inhibitors) are exclusionary, as they would impact the lipid and blood pressure outcomes. Participants may be considered if they are able to consult with their medical provider about temporarily discontinuing these medications long enough for the medication to not be active in the system. Comorbid psychiatric conditions (e.g., depression, personality disorders, substance use disorders) are not exclusionary, as this would result in an unnaturally narrow selection of participants with PTSD. Consistent with standards for intervention studies in PTSD (Mulick and Naugle, 2010; Resick et al., 2002, 2015), those on psychopharmacological medications will be included if the regimen has been maintained as stable for ≥2 months prior to enrollment. This study is approved by the appropriate University Institutional Review Board (IRB). Written informed consent is obtained from all participants at the time of the first outcome assessment (baseline) by the laboratory research assistant(s). As with any human research, a participant may discontinue either intervention condition or any other aspect of the longitudinal study at any time, regardless of the reason.

### Study design

This intervention study examines the effects of a 12-session healthy lifestyle behavior program (focused on physical activity, nutrition, sleep, and stress management) administered in an individual format as an adjunct to usual care psychotherapy (one healthy lifestyle session per week), compared with a usual care psychotherapy-only control condition. The control group will only attend the outcome assessments and usual care psychotherapy sessions. Therefore, the design relies on a between-groups comparison of the two conditions. Referrals for continued care/therapy beyond the study intervention phase are provided by the study team, and participants assigned to the control group are eligible to complete the health intervention after the conclusion of their participation in the control arm.

### Measures

#### Outcome assessment sessions

Four assessment sessions provide an indication of treatment-related outcomes. These sessions are conducted in person at a University-based laboratory at our study site in Ft. Lauderdale, FL (U.S). The first assessment (baseline) is conducted prior to the first week/study onset in each condition. The second assessment (post-treatment) is conducted at the 3-month time point (after the last healthy lifestyle session in the experimental condition). The third and fourth assessments (follow-up) are conducted at the 6-month and 12-month time points. Each assessment session consists of the same outcome measures: blood pressure, lipids, body mass index (BMI), arterial stiffness, endothelial function, heart rate variability (HRV), physical activity, nutrition, perceived stress, sleep (quantity, efficiency, quality, and restfulness), and psychological measures (PTSD symptoms, depressive symptoms). Results of the lipid measures and blood pressure are reviewed with participants following each assessment, and it is recommended to those with clinically elevated levels that they consult with their primary care physician or other medical provider about such findings.

Due to potential confounding effects on lipids and/or blood pressure, participants are asked to refrain from caffeine and strenuous exercise for 3 h, and smoking for 30 min, prior to assessment sessions. These sessions are scheduled early in the morning (approx. 8:30 a.m.) due to the requirement of fasting for lipid assessment. Non-compliance with restrictions requires rescheduling. After the lipid assessment a standardized snack (granola bar, and water/juice) is provided. A doctoral research assistant (under the direction of the PI) leads each assessment session. The data collection for the assessment sessions lasts approximately 90 min. Assessment of the following variables involving physical measurement is conducted first:

#### Fasting lipid profile

The Cholestech LDX System is utilized to assess lipids. The Cholestech is designed for point of service lipid analysis using finger stick capillary whole blood. It uses reflectance photometry to obtain lipid results that are available within 4 min. Lipid values derived from the Cholestech are highly correlated with serum-derived reference values (*r* values 0.96–0.98) (Cobbaert et al., 1993; Polito et al., 2000). Triglycerides, total cholesterol, high-density lipoprotein (HDL), and low-density lipoprotein (LDL) will be measured. Team members who conduct this procedure receive extensive training and practice, including training in precautions with biohazardous wastes. Blood glucose will be considered as an additional variable of interest in secondary analyses.

#### Blood pressure

The participant sits quietly for 5 min, after which three seated, automated systolic blood pressure (SBP) and diastolic blood pressure (DBP) readings (IntelliSense model 5243175; Omron Healthcare Inc.) are taken at 2-min intervals from the left arm to minimize confound with the EndoPat (referenced below for endothelial function and arterial stiffness), for which the right arm is utilized for the test.

#### Body mass

Body weight is measured in pounds with a digital physicians' scale, which permits valid measurement to the nearest tenth of a pound. Height is measured, and the participant's weight is contrasted with height-based norms for the purposes of determining BMI and overweight/obesity status. Measures of waist-to-hip ratio are examined, along with body weight alone, in evaluating body mass/high weight in secondary analyses.

#### Endothelial function

Peripheral arterial tonometry (PAT) signals are obtained using the EndoPAT device (Itamar Medical Inc., Caesarea, Israel), which has been validated and used previously to assess peripheral arterial tone (Halligan et al., 2004; Lavie et al., 2000). EndoPat -derived endothelial function has demonstrated convergent validity with flow-mediated dilation indexed by brachial artery ultrasound scanning (Kuvin et al., 2003) and independent prediction of adverse cardiac events (over 7 years), established endothelial dysfunction, and actual cardiac disease (Bonetti et al., 2004; Rubinshtein et al., 2010). Specially designed finger probes comprise a system of inflatable latex air cuffs connected by pneumatic tubes to an inflating device controlled through a computer algorithm. A constant counter pressure (pre-determined by baseline DBP) is applied through the air cushions. This prevents venous pooling, avoids venoarteriolar reflex vasoconstriction, and there is no arterial blood flow occlusion. Pulsatile volume changes of the distal digit induce pressure alterations in the finger cuff, which are sensed by pressure transducers and transmitted to and recorded by the EndoPAT device. Blood pressure and heart rate are measured using an automated blood pressure monitor (IntelliSense model 5243175; Omron Healthcare Inc.). Endothelial function is measured via an RH–PAT index (Hamburg et al., 2008; Rubinshtein et al., 2010). An RH protocol consists of a 7-min baseline measurement, after which a blood pressure cuff on the right arm is inflated to 60 mmHg above baseline SBP or at least 200 mmHg for 5 min. Occlusion of pulsatile arterial flow is confirmed by the reduction of the PAT tracing to zero. After 5 min, the cuff is deflated, and the PAT tracing is recorded for another 5 min. The ratio of the PAT signal after cuff release compared with baseline is calculated through a computer algorithm, automatically normalizing for baseline signal, and indexed to the contralateral arm. The calculated ratio reflects the RH index.

#### Arterial stiffness

The EndoPat device is also used to assess arterial stiffness. The system calculates peripheral augmentation index (PAIx) from the radial pulsewave analysis (Meeme et al., 2017). PAIx is automatically calculated as the ratio of the difference between the waveform's early and late systolic peaks relative to the early peak (P2–P1/P1), expressed as a percentage. EndoPat arterial stiffness measures have convergent validity with the SphygmoCor device (Ring et al., 2014; Segers et al., 2020), the gold standard for non-invasive central blood pressure and arterial stiffness assessment, and other non-invasive methods (Haller et al., 2007; Perrault et al., 2019). Predictive validity of cardiovascular risk (Framingham), pre-eclampsia, and kidney dysfunction has also been documented for EndoPat arterial stiffness measures (Li et al., 2023; Meeme et al., 2017).

#### Heart rate variability (HRV)

The HRV assessment is also conducted using the EndoPat device via pulsatile volume changes based on the plethysmography waveforms. The frequency domain considers two spectral components and physiological origins: low-frequency (LF; 0.04 to 0.15 Hz; sympathetic modulation) and high-frequency (HF; 0.15 to 0.40 Hz; parasympathetic modulation). The LF/HF ratio reflects the sympathetic balance. The time domain considers the mean of normal sinus rhythm pulse-intervals (MeanNN), the standard deviation of NN-intervals (SDNN), the percentage of successive NN-intervals above 50 ms (PNN50), root-mean-square of successive differences (RMSSD) of NN-intervals, and triangular index (Shaffer and Ginsberg, 2017). SDNN provides an index of total variability, as a function of both parasympathetic and sympathetic influences; RMSSD represents the predominant parasympathetic modulation in cardiac function (Allen et al., 2007). EndoPat HRV measures have been shown to be reliable and to have convergent validity with other non-invasive methods (Linder et al., 2014).

An online computer assessment is utilized for the following self-report measures:

#### Physical activity

The International Physical Activity Questionnaire (7-day long form) is a 27-item measure, developed through international collaboration to be a standard for physical activity assessment. It evaluates time spent in moderate and vigorous activity, walking, and sedentary behaviors over the past week, aligning with current activity guidelines (e.g., 134–135). The IPAQ demonstrates strong test-retest reliability (ICC = 0.66–0.88; Craig et al., 2003) and moderate validity, with correlations of *r* = 0.30–0.50 compared to accelerometer data (Hagströmer et al., 2006). The IPAQ's standardized format ensures consistent and valid assessment of physical activity patterns across diverse populations.

#### Nutrition

Usual dietary intake is assessed using the Food Frequency Questionnaire-Revised (FFQ-R), a widely used and empirically validated tool for estimating habitual dietary intake (Thompson and Subar, 2013). Studies demonstrate its reliability, with test-retest correlations ranging from 0.70 to 0.85, and its validity through significant correlations with both dietary records (*r* > 0.50) and biomarkers such as plasma carotenoids and urinary nitrogen (Subar et al., 2001; Thompson and Subar, 2013; Willett et al., 1985). The FFQ-R is a robust method for efficiently capturing dietary patterns in large-scale studies.

#### Sleep

The Pittsburgh Sleep Quality Index (PSQI) is a self-report survey with 19 items (Buysse et al., 1989) to assess sleep quality and quantity, sleep disturbances, typical time in bed, and sleep efficiency in the past 4 weeks. Responses are used to determine a global score that ranges from 0 to 21, with higher values representing worse sleep quality (5 or greater represents impaired sleep quality). Studies have indicated high sensitivity and specificity for the PSQI in predicting insomnia, high internal consistency, and high test-retest reliability and convergent validity with other sleep measures (Backhaus et al., 2002; Cole et al., 2006).

#### Stress

The Weekly Stress Inventory Short Form (WSI) will be administered to measure self-reported stress. The WSI is a 25-item list of events usually perceived as stressful (Brantley et al., 2007). If the event occurred, participants rate on a 7-pt Likert-type scale the amount of stress caused by the event. The WSI has been found to have adequate internal consistency, reliability, and validity (Brantley et al., 2007).

#### PTSD symptoms

The Posttraumatic Stress Checklist 5th edition (PCL-5) is a 20-item self-report measure that assesses the 20 DSM-5 symptoms of PTSD (Weathers et al., 2013). We will use the version with the Life Events Checklist for DSM-5 (LEC-5) and extended Criterion A component to permit the assessment of trauma events and an index event. Items are rated on a 5-point scale ranging from 0 (not at all) to 4 (extremely) over the past 30 days (Weathers et al., 2013). Good internal reliability, convergent validity with the CAPS, and diagnostic efficiency of the PCL have been demonstrated across trauma populations (Blevins et al., 2015; Wortmann et al., 2016).

#### Substance abuse

The Alcohol Use Disorders Identification Test (AUDIT), a 10-item self-report screening questionnaire, is used to assess alcohol consumption, drinking behaviors, and alcohol-related problems (Barry and Fleming, 1993). The AUDIT demonstrates good reliability and validity across various populations; studies report high internal consistency, with Cronbach's alpha ranging from 0.77 to 0.86 (Barry and Fleming, 1993; Campo-Arias et al., 2013; Daeppen et al., 2000; Zavar et al., 2015). Test-retest reliability is also strong, with correlation coefficients between 0.71 and 0.95 (Daeppen et al., 2000; Zavar et al., 2015). The AUDIT also shows good construct validity, with factor analyses supporting a two-dimensional structure (Campo-Arias et al., 2013; Zavar et al., 2015).

#### Anxiety sensitivity

The Anxiety Sensitivity Index-3 (ASI-3; Taylor et al., 2007) is an 18-item self-report used to assess concern associated with possible negative consequences of anxiety-related symptoms (e.g., “It scares me when my heart beats rapidly”). The scale includes some items from the original ASI (Reiss et al., 1986). Factor analyses consistently support a three-factor structure comprising physical, cognitive, and social concerns (Farris et al., 2015; Kemper et al., 2012; Lim and Kim, 2012; Wang, 2014). The ASI-3 shows good internal consistency, test-retest reliability, and construct validity in various populations, including clinical and non-clinical samples (Farris et al., 2015; Wang, 2014).

#### Experiential avoidance

The Acceptance and Action Questionnaire-II (AAQ-II) is a self-report psychological measure used to assess an individual's level of psychological inflexibility or experiential avoidance (Bond et al., 2011). Higher scores indicate greater levels of experiential avoidance and psychological inflexibility. The AAQ-II demonstrates strong psychometric properties across diverse populations. Multiple studies have confirmed its unidimensional structure, internal consistency, and test-retest reliability (Bond et al., 2011; Lin et al., 2023; Yavuz et al., 2016). The AAQ-II shows concurrent, predictive, and incremental validity in explaining various outcomes, including depression, anxiety, and wellbeing (Bond et al., 2011).

#### Cognitive appraisals

A cognitive appraisal questionnaire developed by Tomaka et al. (1997) is utilized as a self-report instrument designed to measure an individual's cognitive appraisals regarding a specific stressful situation from the past week. Similarly, the Stress Appraisal Measure (SAM) is a self-report instrument comprised of 28 items (Peacock and Wong, 1990); each item is rated on a 5-point Likert scale regarding how the individual feels about the specific stressful situation that was identified in the CAQ; responses range from 1 “Not at all” to 5 “Extremely.” The SAM has been validated across multiple languages and cultures, demonstrating generally satisfactory reliability and validity (Durak and Durak, 2012; Durak and Senol-Durak, 2013). Convergent and discriminant validity have been established through correlations with related measures (Durak and Senol-Durak, 2013). The SAM's ability to predict stress-related outcomes and its gender invariance have also been supported (Durak and Durak, 2012; Durak and Senol-Durak, 2013).

#### Depressive symptoms

The Beck Depression Inventory-2nd Ed. (BDI-II) is a 21-item self-report measure assessing depressive symptoms, including suicidal ideation, over the past 2 weeks (Beck et al., 1996). It demonstrates high reliability, with internal consistency coefficients (α = 0.89–0.93) and strong test-retest reliability (*r* = 0.73–0.96) (Beck et al., 1996; Dozois et al., 1998). Its validity is supported by correlations with other depression measures, such as the Hamilton Depression Rating Scale (*r* = 0.71) and PHQ-9 (*r* = 0.67), and its sensitivity to changes in symptom severity (Beck et al., 1996).

#### Health-related self-efficacy

The Health-Specific Self-Efficacy Scales measure individuals' confidence in their ability to successfully engage in behaviors related to preventive nutrition, physical exercise, and alcohol resistance (Schwarzer and Renner, 2005). These scales are grounded in social-cognitive theory and assess perceived self-efficacy, which refers to one's belief in one's capacity to perform actions necessary to achieve desired health outcomes (Schwarzer and Renner, 2000). Each scale includes items addressing barriers to behavior change, such as managing routines or coping with stress. The scales demonstrate strong reliability, with Cronbach's alpha values of 0.87 for the nutrition scale, 0.88 for the physical exercise scale, and 0.79 for the alcohol resistance scale, indicating high internal consistency (Schwarzer and Renner, 2005). Evidence supports the scales' utility in predicting health-related intentions and behaviors, with significant correlations between self-efficacy scores and subsequent behavior changes over time (Schwarzer and Renner, 2005).

#### Demographic measures

Basic demographic information, including age, race, ethnicity, family income, marital status, and education level, is assessed by self-report.

#### Medication use

Medication use is assessed by self-report during the screening period to determine participant eligibility, ensuring that current medications do not confound study outcomes. At each lab assessment time point, participants are asked to report any changes in medication use. An open-response option is provided to capture detailed information, allowing participants to type in the names and dosages of their specific medications. This approach enables comprehensive and precise documentation of medication use across all study phases.

#### Accelerometry

Participants are provided with an Actigraph wGT3X-BT watch and instructed on its use to track accelerometer-based physical activity and sleep measures each day for 1 week following each assessment session. After the watch is returned, data are downloaded for later analysis. The wGT3X-BT is the industry standard for accelerometer monitoring of physical activity and sleep (Breteler et al., 2019; Romanzini et al., 2014). The device is small (weighing only 19 grams), unobtrusive, tamper-resistant, well-tolerated, and does not hinder activity (Breteler et al., 2019). The accompanying ActiLife software is utilized for data download and analysis.

### Procedures

#### Recruitment and enrollment

Participants are recruited from university and community-based clinics, private practices, and from the community using written and online notices/flyers. Locations for recruitment within the community using written flyers include community college campuses, bus/train stops, parking garages, public bulletin boards, public libraries, public restrooms, convenience stores, shopping centers/grocery stores with community bulletin boards, laundromats, religious centers (churches), community centers, public parks, and recreation centers. Online notices for the study are posted on Craigslist, Facebook, and Instagram. Participants are also recruited by other participants within the study. Potential participants call a private central phone number or respond to the study email address to express interest in the study. Volunteers are then screened by phone to assess basic study inclusion and exclusion criteria and to provide additional details about the study. Randomization to study condition is performed by the project coordinator following the initial phone contact (using the flip of a coin). Neither the participants nor the investigators are blinded for this trial. At the baseline/pre-intervention assessment, informed consent and additional screening assessments are administered to further evaluate and verify eligibility based on PTSD symptoms and the presence of at least one risk factor targeted by the intervention (e.g., physical inactivity, overweight status, sleep difficulties). All records are kept confidential. A list of participant names linked to their code numbers is kept in a secured file and accessed by study coordinators for project-related communications. Only code numbers are used to organize participant study data. The coded project data are kept in a separate secured file. Participant compensation consists of a $50 gift card for each of the pre-, post-, and follow-up assessment sessions, and a $10 gift card for each intervention session completed by the participant.

#### Training of study interventionists

The interventionists for the project are advanced doctoral students with experience delivering CBT-based health interventions and/or trauma-focused therapies (depending on the protocols they are trained to conduct). The health behavior interventionists are familiar with the healthy lifestyle intervention literature and develop a thorough understanding of the protocol for PTSD patients by reviewing the manual and meeting with the PI for training. Interventionists are encouraged to meet with the PI throughout intervention delivery to affirm their knowledge and confidence with the protocol and address any questions that might relate to participant-specific presentations.

For interventionists who conduct the standard trauma therapies, the PI reviews evidence-based conceptual and intervention models of trauma-focused treatment for PTSD, including Cognitive Processing Therapy (Resick et al., 2002; Resick and Schnicke, 1992), Prolonged Exposure Therapy (Foa et al., 2007; McLean and Foa, 2024), and as applicable for participants with a presentation consistent with the literature on complex-PTSD, Contextual Trauma Therapy (Gold and Quiñones, 2020). Trauma therapists are encouraged to review treatment-specific readings for the various models of treatment reviewed during training and meet with the PI and project coordinators (designated as lead interventionists) to discuss treatment options for assigned participants. Trauma therapists select treatment approaches based on the clinical presentations of participants and collaboration with participants about the various options for care. Given the literature suggesting increased rates of drop-out in exposure-based treatment, it was imperative that trauma therapists have a strong rationale for this approach, a deep understanding of its implementation, and the ability to educate the participant about the treatment delivery (Imel et al., 2013). Trauma therapists are invited to discuss their approaches to treatment, ask questions about intervention implementation and participant clinical presentations, as well as discuss relevant research on PTSD treatment during monthly research lab meetings, in one-on-one meetings with the PI, and in peer supervision meetings with the research coordinators.

#### Health behavior/lifestyle intervention

The healthy lifestyle intervention is a 12-session program with sequential but overlapping modules for increasing physical activity, enhancing good nutrition, improving sleep, and improving stress management. The sessions are conducted individually and are held weekly for 90 min. Participants have the option of conducting the intervention sessions (health behavior and/or trauma therapy sessions) in-person or virtually via a secure HIPAA-compliant Zoom platform. The length of the program was determined by the need to be long enough to effectively address the 4 modules, while also being relatively brief to minimize burden for participants and enhance likelihood of compliance. For the current project, the decision to utilize 12 sessions was also informed by positive feedback from the participants and interventionists in the pilot study regarding the program length.

The first module includes psychoeducation on physical activity (health benefits of physical activity and the health risks associated with physical inactivity and overweight) in session 1, as well as implementation of an exercise regimen and discussion of some of the inherent challenges. Participants are encouraged to begin a physical activity regimen that is consistent with recommendations for a healthy lifestyle (aerobic exercise at least 20 min 2–3 times per week). However, interventionists instruct participants to work toward exercise goals that can be incorporated into their lifestyles and maintained. In addition, participants walk for 20 min with the interventionist during intervention sessions 2–5. Session 2 includes discussion of healthy food choices, health risks associated with unhealthy foods, appropriate caloric intake for weight loss (as applicable), reading food labels, fat, vitamin, and sugar content, and setting dietary goals. The interventionist also helps participants distinguish diets from lifestyle changes, emphasize self-monitoring of foods, and helps participants build strategies for nutritional self-control. Nutritional recommendations include a balanced diet, enhancing consumption of fruits and vegetables, and reducing sugars, fat/saturated fat, and caloric intake (as appropriate per participant goals). During sessions 3 and 4, the participant and interventionist work together to analyze current nutritional habits and plan a healthier nutritional plan. Session 3 includes discussion of participant efforts to incorporate physical activity and healthy nutrition into their lifestyle, and the potential for social support to be helpful in behavior change. This session also addresses stress and eating. Interventionists closely monitor the stress coping skills of participants and discuss potential distress associated with giving up previously used unhealthy eating approaches to cope (especially in coping with PTSD symptoms). To address potential desire to be overweight for protection against abuse, alternative methods of achieving a sense of safety are discussed. Session 4 focuses on the skills of shopping for healthy food, planning healthy meals, and reducing behavioral cues/triggers for binge eating and unhealthy eating (e.g., storing foods out of sight, strategies for parties, special events and restaurants, managing emotions). Sessions 5 and 6 will include further discussion of progress and challenges with the physical activity and nutrition regimens.

The sleep-focused intervention begins in session 5 and continues into session 8. In the fifth session, an overview of common sleep problems is introduced, and the participant addresses any sleep difficulties they have had. A rationale for the treatment used in our protocol is provided. During sessions 6 through 8, cognitive behavioral strategies for assisting with sleep improvement are incorporated and discussed. In session 6, participants also identify a problematic nightmare (where applicable) and initiate the process of imagery rehearsal therapy by beginning to develop and write a modified version of the dream. In addition to discussion of insomnia treatment strategies in sessions 7 and 8, participants continue to develop their modified dream and rehearse it in session. Participants are also instructed to rehearse the modified dream daily for 5–20 min.

Sessions 9–12 focus on stress management, relapse prevention, and other issues related to termination of the program. During session 9, the interventionist provides an overview of the cognitive-behavioral model of stress and initiates a discussion of various aspects of the model (e.g., negative thoughts, negative emotions and their relationship to behavior). Between sessions 9 and 10, participants are asked to self-monitor negative thoughts to provide a basis for follow-up discussion in session 10, which focuses on common patterns of negative thoughts and associated negative feelings. During session 10, participants are also familiarized with thought logs (Beck, 2021), which use personal situations identified by participants to assist in identifying relationships between negative thoughts, negative feelings, and behavioral outcomes, as well as constructing responses to stressful situations that are more adaptive. Participants are given homework to complete two or more thought logs between sessions 10 and 11, and the results are discussed during session 11. During session 12, discussion of thought logs and stress coping is continued for the first part of the session. The last portion of the final session includes a review of the skills learned, discussion of risks for relapse and skills for relapse prevention, and discussion of any remaining concerns associated with termination of the intervention program.

### Adherence and strategies for retention

Participants randomized to the experimental (health behavior intervention) arm of the proposed project have the potential to be helped by the program in terms of their physical and mental health. It is expected that the health behavior program and the opportunity to receive free trauma therapy on a time-limited basis are elements that enhance retention (in addition to the gift card incentives). However, given that the study's objective is to determine the effectiveness of the experimental intervention, it is not possible to estimate the extent to which direct benefits will occur. At each assessment session, participants also receive educational materials about their cardiovascular risk assessment results (e.g., # of risk factors, lipid profile, blood pressure).

### Power analyses, data reduction and statistical analyses

The following effect sizes for power calculations are based on studies that included outcome variables that are of central relevance to our intervention and most closely approximated the methods for the present study.

#### Physical activity and BMI

A study of brief CBT intervention for increasing exercise and reducing BMI in a sample with serious mental illness (Brown and Chan, 2006) demonstrated an increase in exercise for the intervention compared with no change for controls [large effect (Cohen's *d* = 0.89)]. There was a significant difference in BMI change for the intervention compared with controls (*d* = 1.19). Our pilot data are consistent with these findings; thus, we estimate a large effect for improvement in physical activity (*d* = 0.70–0.89).

#### Nutritional consumption and lipid levels

Based on the finding of improved nutrition in response to a computer-based nutritional intervention relative to a control group (O'Brien and Palfai, 2016), we calculated a medium effect size (*d* = 0.54). Based on findings of improved lipids in response to a community-based weight management and nutrition program (Graffagnino et al., 2006), we calculated a moderate effect (*d* = 0.50). This study is consistent with a qualitative review (Laederach-Hofmann et al., 2008) that indicated long-term positive effects of weight management on lipids.

#### Sleep intervention

In an RCT of 168 sexual assault victims with PTSD, IRT resulted in large improvements in number of nights with disturbing nightmares (d = 1.24) and total number of weekly nightmares (*d* = 1.27), and a medium effect for improved sleep quality (*d* = 0.67), compared with waitlist (Krakow et al., 2001). Our pilot data indicate a large effect for improved sleep quantity (*d* = 1.41). Therefore, we estimate effects for sleep quantity and quality outcomes to range from 0.67 to 1.41.

#### Improved blood pressure

A study that examined the effects of an exercise program on blood pressure (Morris et al., 2021) evidenced a moderate effect for SBP (*d* = 0.60) and a large effect for DBP (*d* = 0.83). These findings are consistent with systematic reviews (Aucott et al., 2009; Laederach-Hofmann et al., 2008) that indicate long-term positive effects of weight loss studies on BP. Our pilot data supports a moderate effect for DBP in the PTSD population (*d* = 0.51).

#### Improved CVD markers

In a meta-analysis examining HRV in PTSD, a small subset of studies was identified that examined changes in HRV as a treatment outcome (Nagpal et al., 2013). A range of moderate to large effects was evident for the respective studies and measures of HRV, with a large overall effect (*d* =1.89). To date, we are not aware of any published studies in the PTSD population that have attempted to intervene on arterial stiffness or endothelial function. Therefore, our effect size estimates come from a broader range of studies that examined behavioral interventions with these outcomes. Cavero-Redondo et al. (2021) conducted a meta-analysis of exercise interventions and the effects on arterial stiffness, with an overall moderate effect (*d* = 0.56). A meta-analysis of exercise studies and endothelial function (Lee et al., 2018) indicated that low-moderate intensity aerobic exercise produced desirable improvements in endothelial function (*d* = 0.55).

#### Summary

Completing 162 protocols (81 per condition) with minimum power of 80% and alpha level of 0.05 will permit detection of a moderate effect of 0.47 or greater (Cohen, 1988). Therefore, this sample size would permit detection of the smallest effect estimated above for lipids (*d* = 0.50) and will be conservative for the projected large effects for some of the estimates above. Participants in the health behavior intervention condition (experimental group) are considered treatment completers if ≥75% of sessions are attended.

#### Data analysis plan

Preliminary analyses will be conducted to examine distributions, detect outliers, and compare the study groups on demographic variables, comorbid psychopathology, type of trauma therapy received, smoking, and alcohol use. Based on the randomization, no differences for demographic (e.g., age, gender) or other baseline comparison variables are expected. In addition, gender is not predicted to interact with the main study effect (condition). If there are group differences in any of the baseline comparison/control variables, the respective variable(s) will be treated as a control in the analyses.

A between-groups approach is used, with study condition as the independent variable (IV) and changes in the outcomes as the dependent variable(s) (DVs). The outcomes will be analyzed using linear mixed-effects regression. This approach is recommended for between-group longitudinal trials with follow-up time points to model changes over time, to incorporate all available data at each time point, and to account for non-independence of observations inherent in repeated-measures data (Verbeke and Molenberghs, 2000). A piecewise model will be specified to model change during distinct time periods of the trial (Raudenbush and Bryk, 2002). Separate slopes will be provided for the pre- to post-treatment phase, and each follow-up point (pre- to 6-month follow-up, and pre- to 12-month follow-up). Multivariate analyses will be used for each hypothesis to control for type 1 error by testing the DVs together. Significant multivariate effects will be followed with univariate tests.

## Discussion

The presently described project examines the potential positive health effects of a relatively short-term healthy lifestyle intervention for adults with PTSD and CVD risk. In a pilot study, our health behavior intervention program had the greatest impact on sleep and physical activity (Kibler et al., 2023). Given the prior data on sleep disruption and lower physical activity in PTSD (Harte et al., 2015; Lamarche and De Koninck, 2007), such outcomes in the present study would be considered major changes that would likely enhance health and quality of life if changes are maintained for individuals with PTSD.

There is preliminary evidence that simultaneous physical activity and nutritional intervention may be more effective for impacting eating habits than focusing on the respective behaviors sequentially (Johnson et al., 2014; Vandelanotte et al., 2005). Overlapping modules in the proposed program will encourage building skills for a healthier lifestyle early in the intervention. In addition, focusing on multiple health behaviors at once may permit participants' detection of interacting variables (e.g., a participant does not sleep well on days that they do not exercise) (Johnson et al., 2014; Prochaska et al., 2008). Physical activity and nutrition are emphasized in sessions 1–5; participants are instructed to continue implementing their physical activity and nutritional plans throughout the intervention, and to continue these regimens beyond completion of the program. Our efforts to address sleep in conjunction with other health behaviors may also confer a synergistic effect that is greater than the impact of each component alone (Nagaya et al., 2007; Prochaska et al., 2008). One study showed a positive association between exercise intervention and improved sleep for individuals with PTSD (Björkman and Ekblom, 2021).

Overall, the present project will provide essential data to evaluate whether a health behavior program can produce consistent improvements in stress that exceed standard care for adults with PTSD. There are several methodological strengths that permit a thorough evaluation of changes in health risks over time. Including a 9-month longitudinal follow-up period permits better detection of longer-term changes in stress and other health-related outcomes. The use of objective sleep and physical activity monitoring using the ActiGraph technology provides a stronger basis for evaluating the changes in these parameters than self-report alone for the present study (Eid et al., 2021).

In conclusion, the present study entails an extensive evaluation of cardiovascular health risks in adults with PTSD. It provides a valuable assessment of the effects of a health behavior intervention designed specifically for this population. The study results will inform multidisciplinary and/or multicomponent intervention that may have a significant impact on quality of life for patients with PTSD.

## Ethics and dissemination

### Potential risks

Participation in the proposed study poses some psychological and physical risks associated with the assessment and treatment procedures. Assessment and interventions with individuals who have PTSD can elicit negative emotions. Some of these emotions may be newly experienced. The participant may feel that discussion of traumatic experiences makes them feel worse in the short-term. Over the long-term, discussing traumatic events and the emotional sequelae has been found to be clinically beneficial.

For the lipid assessment, a finger stick is performed. Possible risk associated with the finger stick may be discomfort. Less commonly, infection may occur. There is also potential risk associated the exercise regimens and/or any dietary changes that participants commence. If a participant has any medical reason that might make exercise dangerous, participation could pose a health risk. We advise that each participant consult with a physician before making any changes to their physical activity or diet.

### Protection against risks

At the baseline assessment, written informed consent is sought by the advanced doctoral student assistant, who verbally evaluates the participant's understanding of the study and consent form. All participants are carefully monitored throughout all assessment procedures. The PI ensures proper clinical care to study candidates who appear to be experiencing strong emotional reactions during any part of the study, and works with the project staff to help participants who require additional assistance coordinating care. Because depressive symptoms commonly co-occur with PTSD symptoms, participants are monitored at each assessment session for depressive symptoms and suicidality via the BDI-II. Experimenters/interventionists are trained to assess suicide lethality and are provided with specific steps to follow in the event that a participant is actively suicidal. Clinic support staffs are available to assist with a crisis during the assessments. The experimenter will be instructed to contact the PI when a participant experiences active suicidal intent, and appropriate clinical coverage (licensed psychologist) is designated at any time that the PI is not available. Non-lethal suicidal ideation is handled in the manner described above, with the study staff and PI ensuring adequate clinical follow-up of such symptoms.

To minimize the risk of infection for the finger stick, universal precautions for sanitary collection and handling of human blood samples are followed. In addition, participants are free to decline any procedure that may be distressing for them, and/or withdraw from the study at any time. Therefore, participants may decline or terminate the finger stick procedure at any time. To minimize the risk associated with the exercise intervention, participants are advised to get physician clearance for participation in an exercise regimen.

All records are kept confidential. Only code numbers are used to organize participant data. The coded project data will be kept in separate secured file. No information about the participant will be released to outside parties without consent. Under certain circumstances, records may be reviewed for audit purposes by authorized University employees who will be bound by the above described provisions of confidentiality. No identifying information will be included in any project presentations or publications.

### Potential benefits to participants and others

Participants randomized to the experimental (healthy lifestyle intervention) arm of the proposed project have the potential to be helped by the program in terms of their physical and mental health. However, given that the objective of the study is to determine the effectiveness of this experimental intervention, it is not possible to estimate the extent to which this will occur. All participants receive educational materials about their cardiovascular risk assessment results (e.g., # of risk factors, lipid profile, BP) at each assessment session. Given the care and attention to minimizing risks in this study, we believe the risks are reasonable in relation to the benefits.

### Importance of knowledge to be gained

The knowledge gained from this research could be extremely valuable. The rates at which adults with PTSD experience CVD and cardiovascular risk factors represent a significant public health problem. The need for developing effective interventions for CVD risk-reduction in PTSD is increasingly evident (as discussed in further detail in the Specific Aims and Significance sections of the proposal). The present project addresses several gaps in the previous research on PTSD and CVD reduction; the project will be critical in evaluating whether adjunctive treatments, such as health behavior interventions, may be necessary as supplements to traditional psychotherapy for PTSD in order to reduce CVD risks. Thus, the project has the potential to inform intervention efforts which extend beyond the current standard of care for PTSD. Given the knowledge that may be gained from this project, we believe the minimal risks to participants are justifiable.

### Dissemination

We have registered this clinical trial with ClinicalTrials.gov, as outlined in the NIH Policy on the Dissemination of NIH-Funded Clinical Trial Information. The PI will submit summary results information no later than 1 year after the trial's completion date. We will comply with all terms and conditions of the NIH award. The University has an internal policy to ensure that awardees comply with the NIH Policy on the Dissemination of NIH-Funded Clinical Trial Information.
